# Safety Concerns in Mobility-Assistive Products for Older Adults: Content Analysis of Online Reviews

**DOI:** 10.2196/42231

**Published:** 2023-03-02

**Authors:** Namrata Mali, Felipe Restrepo, Alan Abrahams, Laura Sands, David M Goldberg, Richard Gruss, Nohel Zaman, Wendy Shields, Elise Omaki, Johnathon Ehsani, Peter Ractham, Laddawan Kaewkitipong

**Affiliations:** 1 Department of Computer Science Virginia Tech Blacksburg, VA United States; 2 Department of Industrial and Systems Engineering Virginia Tech Blacksburg, VA United States; 3 Department of Business Information Technology Virginia Tech Blacksburg, VA United States; 4 Center for Gerontology Virginia Tech Carilion School of Medicine Blacksburg, VA United States; 5 Department of Management Information Systems San Diego State University San Diego, CA United States; 6 Department of Management Radford University Radford, VA United States; 7 Department of Management, Information Systems & Quantitative Methods The University of Alabama at Birmingham Birmingham, AL United States; 8 Department of Health Policy and Management Bloomberg School of Public Health Johns Hopkins University Baltimore, MD United States; 9 Center of Excellence in Operations and Information Management Thammasat Business School Thammasat University Bangkok Thailand

**Keywords:** injury prevention, consumer-reported injuries, older adults, online reviews, mobility-assistive devices, product failures

## Abstract

**Background:**

Older adults who have difficulty moving around are commonly advised to adopt mobility-assistive devices to prevent injuries. However, limited evidence exists on the safety of these devices. Existing data sources such as the National Electronic Injury Surveillance System tend to focus on injury description rather than the underlying context, thus providing little to no actionable information regarding the safety of these devices. Although online reviews are often used by consumers to assess the safety of products, prior studies have not explored consumer-reported injuries and safety concerns within online reviews of mobility-assistive devices.

**Objective:**

This study aimed to investigate injury types and contexts stemming from the use of mobility-assistive devices, as reported by older adults or their caregivers in online reviews. It not only identified injury severities and mobility-assistive device failure pathways but also shed light on the development of safety information and protocols for these products.

**Methods:**

Reviews concerning assistive devices were extracted from the “assistive aid” categories, which are typically intended for older adult use, on Amazon’s US website. The extracted reviews were filtered so that only those pertaining to mobility-assistive devices (canes, gait or transfer belts, ramps, walkers or rollators, and wheelchairs or transport chairs) were retained. We conducted large-scale content analysis of these 48,886 retained reviews by coding them according to injury type (no injury, potential future injury, minor injury, and major injury) and injury pathway (device critical component breakage or decoupling; unintended movement; instability; poor, uneven surface handling; and trip hazards). Coding efforts were carried out across 2 separate phases in which the team manually verified all instances coded as minor injury, major injury, or potential future injury and established interrater reliability to validate coding efforts.

**Results:**

The content analysis provided a better understanding of the contexts and conditions leading to user injury, as well as the severity of injuries associated with these mobility-assistive devices. Injury pathways—device critical component failures; unintended device movement; poor, uneven surface handling; instability; and trip hazards—were identified for 5 product types (canes, gait and transfer belts, ramps, walkers and rollators, and wheelchairs and transport chairs). Outcomes were normalized per 10,000 posting counts (online reviews) mentioning minor injury, major injury, or potential future injury by product category. Overall, per 10,000 reviews, 240 (2.4%) described mobility-assistive equipment–related user injuries, whereas 2318 (23.18%) revealed potential future injuries.

**Conclusions:**

This study highlights mobility-assistive device injury contexts and severities, suggesting that consumers who posted online reviews attribute most serious injuries to a defective item, rather than user misuse. It implies that many mobility-assistive device injuries may be preventable through patient and caregiver education on how to evaluate new and existing equipment for risk of potential future injury.

## Introduction

### Background

The Consumer Product Safety Commission (CPSC) has reported that from 2016 to 2020 there were approximately 14.6 million emergency department (ED) cases associated with older adults using consumer products [1]. Of these, nearly two-thirds were due to falls [2], indicating that the dominant context of injury is during motion or transfer of older adults. Mobility devices such as canes and walkers are among consumer products frequently implicated in such injuries. The development of interventions to prevent potential future injuries among older adults using mobility-assistive devices requires a better understanding of equipment limitations or failures that precipitate injuries from using such devices. A substantive curated data set of safety concerns regarding mobility-assistive devices would address this gap in knowledge.

The adoption of these devices is relatively common, with approximately 10% of community-living adults aged ≥65 years adopting them each year to help with their mobility limitations [3]. Given that older adults are among the fastest growing demographic groups in the United States [4], annual injury figures are expected to continue increasing within the next decade. In fact, the Centers for Disease Control and Prevention anticipates that by 2030 there will be 7 older adult fall-related fatalities per hour [5]. When compared with an estimated 4.17 fall-related fatalities per hour in 2020 [6], this calls for expanded injury prevention mechanisms capable of rapidly detecting safety concerns regarding products targeted toward older adults.

Regulatory agency injury prevention efforts often rely on customers and businesses to report unsafe products because it is logistically impossible to inspect every product in the market. It should be noted that safety hazards are generally reported after consumer harm, failing to prevent injury; for example, between 2014 and 2021, the CPSC issued recalls for hundreds of thousands of units of 4 bed handle brands but only after multiple deaths were attributed to these products [7-11]. Although existing databases such as the CPSC’s National Electronic Injury Surveillance System (NEISS) and the Food and Drug Administration’s Manufacturer and User Facility Device Experience (MAUDE) contain reports of consumer harm caused by product failure, these are generally abstracted from a third party, not the consumer itself, which poses several problems. In the case of NEISS, because data are abstracted from ED visit records, the content is highly skewed toward injury description, and context regarding how the device failed is scarce and ambiguous (eg, “the walker broke”). Regarding MAUDE, although it does abstract from various sources (hospitals, device user facilities, voluntary reports, and manufacturers), report-processing times may result in untimely reports of product malfunctions. Moreover, it should be noted that consumers may often forgo voluntary reporting to the Food and Drug Administration because they may believe that the issue is “not that serious,” when it could, in fact, be a malfunction capable of causing serious injury. Regarding MAUDE manufacturer reports, these are only required if there is reasonable information suggesting that the product may contribute to consumer death or serious injury, and, as a result, such reports are rare. Thus, given the long delays in reporting, investigating, and initiating regulatory action for product safety hazards, as well as existing database content tending to focus on circumstances other than device malfunction context, additional resources are needed to inform older adults and their caregivers about the safety of mobility-assistive devices.

Although medical device stores, specialized pharmacies, and physiotherapists may provide warnings or recommendations on specific devices, access to these may not be straightforward for every older adult. Location or health concerns may prevent access, leading to an older adult demographic that relies on online retailers for purchase of these devices. In this regard, online reviews are often used by consumers and regulators to assess the safety of products. Studies show that 0.2% to 4% of online reviews contain evidence of a product safety concern [12-15] that cannot be found in reports by regulatory agencies. The early work by Restrepo et al [16] indicates that online product reviews present a novel and untapped source of safety concerns for mobility-assistive devices for older adults. As the thousands of available reviews for any given device may be overwhelming and difficult to navigate for an older adult, further work is needed to substantially expand upon, analyze, and compile consumer-reported safety concerns of mobility-assistive devices.

### Objectives

The aims of this study were to investigate older adult injuries stemming from the use of mobility-assistive devices, as reported by older adults or their caregivers. We not only identify injury severities and mobility-assistive device failure pathways but also shed light on the development of safety information and protocols for these devices. To do so, we developed and used an expansive, manually curated data set of mobility-assistive device safety concerns from consumer reviews on Amazon’s US website. On the basis of the findings from our prior studies, we expect that the curated data set of online reviews for mobility-assistive devices will provide valuable insight into the types of injuries that are associated with the use of mobility-assistive devices and the pathways by which mobility-assistive device use precipitates injuries.

## Methods

### Data Source

Initial data were extracted from Amazon’s US website through the use of an automated script [16]. The script extracted 633,141 reviews, distributed across thousands of unique products pertaining to “assistive aid” categories typically intended for older adults. The reviews were collected from September 12, 2017, to September 16, 2017. The reviews span the time range from February 28, 2002, to September 15, 2017.

### Exploratory Data Coding

Large-scale data coding (labeling) efforts were undertaken as volunteers became available, resulting in 2 separate exploratory coding phases (I and II), described later in this section, and visualized in Figure S1 and Table S1 in Multimedia Appendix 1 [17]. Coding categories were iteratively refined by having senior raters, who were the lead authors (all having graduate student or faculty investigator status), code random samples and discuss discrepancies across both phases. Cohen κ [17] was used to verify interrater reliability (scores presented in Table S1 in Multimedia Appendix 1). Throughout both phases, junior raters, who were undergraduate student volunteers who had completed the qualification examination verifying that they were able to code safety concerns with an accuracy of ≥80%, were asked to code reviews according to injury type:

- Major injury occurred: someone was seriously hurt by the product, as described in the following review, and required a physician or hospital visit (medical professional intervention) or died:

Very unstable as wheelbase is way too short...product [went] from under me walking down a ramp at least 6 times...broke my thumb.

- Minor injury occurred: someone was actually hurt by the product, but it was a minor, self-treatable incident, and no physician visit or hospital visit was explicitly mentioned:

Seems to be not very stable, my mother fell with it and she really scraped her leg badly...she doesn’t want to use it.

- Potential future injury: injury could possibly occur; thus, the review writer is cautious about using the product:

Not even 3 months old and already the wheels are falling off! my husband is handicapped and would not be good if the walker collapsed when he tried to sit because the wheel came off!

- No injury occurred: there is no indication of an actual or potential future injury as a result of product use:

Sturdy cane. The stands by itself feature, very nice.

Phase I, described in Restrepo et al [16], encompassed the coding and examination of 50,000 randomly selected reviews, which resulted in the identification of 18 major product categories (Table S2 in Multimedia Appendix 1) and 3100 (6.2%) shortlisted reviews of potential safety concerns. Shortlisted concerns pertaining to categories unrelated to mobility-assistive devices were removed, and of the 3100 reviews, we retained only 983 (31.7%). Of the 18 major product categories, we retained only 8 (44%; Table S2 in Multimedia Appendix 1).

Before phase II coding, the 8 retained categories were further narrowed down to 5 (63%): *canes, gait and transfer belts, ramps, walkers and rollators,* and *wheelchairs and transport chairs*. *Crutches* as well as *knee walkers and scooters* were determined to be frequently used by younger adults (aged 25-64 years), rather than predominantly older adults (aged ≥65 years), and *car assistive devices* were re-examined and found to be unrelated to actual mobility assistance, resulting in the elimination of these 3 categories. Phase I verified concerns were filtered accordingly: of the initial 983 reviews, we preserved 685 (69.7%). In addition, the 633,141 review sample pool was reduced to 44,119 (7%), solely keeping reviews (not previously labeled in phase I) belonging to the 5 retained mobility-assistive device categories. Table 1 displays mobility-assistive device review distributions for each of the phases.

The 44,119 retained reviews were coded by 190 undergraduate student volunteers (junior raters) from a triple crown–accredited university in Thailand and an R1 public land-grant university in the United States, which resulted in 3300 (7.48%) shortlisted online reviews that mentioned potential safety concerns. Volunteer work was validated by methods described in phase I [16], in which 2 of the authors and a doctoral student (senior raters) verified both whether the concern was relevant and what the nature of the concern was, resulting in, out of the 3300 shortlisted online reviews, a final list of 2203 (66.76%) online reviews that mentioned safety concerns. The Cohen κ [17] value among the senior raters for the final list was 0.84.

A mobility-assistive device safety concern data set comprising 2888 reviews was created by consolidating the validated concerns of both phase II (n=2203, 76.28%) and phase I (n=685, 23.72%).

**Table 1:** Phases I and II volunteer-coded mobility-assistive device review pool distribution.

Category (Amazon’s US website) and focal product type		Unique products (n=487), n (%)	Phase I reviews (n=4767), n (%)	Phase II reviews (n=44,119), n (%)	Total reviews (n=48,886), n (%)
**Health and household**					
	Canes	179 (36.8)	2037 (42.7)	15,664 (35.5)	17,701 (36.2)
	Gait and transfer belts	31 (6.4)	106 (2.2)	1524 (3.5)	1630 (3.3)
	Ramps	47 (9.7)	160 (3.4)	2091 (4.7)	2251 (4.6)
**Mobility aids and equipment**					
	Walkers and rollators	119 (24.4)	1301 (27.3)	14,987 (34)	16,288 (33.3)
	Wheelchairs and transport chairs	111 (22.8)	1163 (24.4)	9853 (22.3)	11,016 (22.5)

### Additional Data Coding

The manual validation efforts of phases I and II led to the discovery of 7 device failure mechanisms (injury pathways), resulting in additional coding (of the 2888 reviews) by the authors. Coding disagreements were discussed by the authors to refine the 7 identified injury pathways, leaving 6 (86%) discrete categories (pathways):

- Critical component breakage or decoupling: defined as the device or a part or parts of it breaking, falling off, frequently requiring adjusting or retightening (with the exception of brakes), or collapsing; also used when the device was described as being cheaply made, “unsturdy,” or flimsy
- Unintended movement: defined as the device or a part or parts of it moving without the user intending it to, although the part itself is not generating instability or at risk of decoupling; also used when the device’s brakes were said to require frequent adjusting or retightening
- Instability: defined as the device or a part or parts of it causing instability or unsteadiness while being used; also used when the device was described as wobbly, tippy, off-balance, insecure, or unable to provide adequate support
- Poor, uneven surface handling: defined as the device or a part or parts of it frequently getting caught or stuck in small cracks or seams, tangled in grass or gravel, or unable to cross small bumps or thresholds; also used when the device was explicitly described as unsafe when used outside
- Trip hazards: defined as the device or a part or parts of it being prone to tripping the user; also used when the device was described as frequently catching the user’s feet
- Design failure: defined as the device or a part or parts of it failing to safely assist the user because of a clearly identifiable design flaw not described by the previous 5 pathways (eg, poor ergonomic design, brake accessibility, or absence of brakes [by design]).

Of the 2888 reviews, 32 (1.11%) were deemed to provide insufficient context surrounding the injury to be accurately classified, and 128 (4.43%) had concerns related to shipping and inspection quality (missing parts, broken upon arrival, etc) rather than true safety concerns. Thus, of the 2888 reviews, 160 (5.54%) were removed, leaving 2728 (94.46%).

### Statistical Methods

No hypotheses were specified a priori; therefore, no statistical tests were performed. The values presented in the following tables represent the normalized count per 10,000 reviews of the relevant product categories.

### Ethical Considerations

Our research collected and analyzed secondary data from publicly available reviews on Amazon’s US website. The reviewers’ names were not included in the analysis. Reviews are posted on a public forum, where reviewers do not have an expectation of privacy. This type of research was therefore classified as exempt regarding institutional review board considerations. Our research did not involve direct personal interaction with any human participants.

## Results

### Injury-Type Distributions

Mobility-assistive device injury–type distributions and totals as well as sample cases are presented in Table 2 and Table 3, respectively. Major-injury distributions ranged from 0 per 10,000 reviews (ramps and wheelchairs or transport chairs) to a high of 10 (0.1%) per 10,000 reviews (walkers or rollators). Minor-injury distributions ranged from 22 (0.22%) per 10,000 reviews (ramps) to 57 (0.57%) per 10,000 reviews (wheelchairs or transport chairs). When combined with major injuries, per 10,000 reviews, 240 (2.4%) voice hazardous experiences entailing *actual* user injury. As for potential future injury, gait or transfer belts had the lowest count (344 instances per 10,000 reviews, 3.44%), suggesting that users may be at increased risk of actual injury while using a defective gait belt. By contrast, canes had the highest potential future injury count (614 instances per 10,000 reviews, 6.14%).

**Table 2:** Mobility-assistive device by injury type per 10,000 reviews.

Mobility-assistive device	Major injury, n (%)	Minor injury, n (%)	Potential future injury, n (%)
Cane (n=672)	2 (0.3)	56 (8.3)	614 (91.4)
Gait or transfer belt (n=393)	6 (1.5)	43 (10.9)	344 (87.5)
Ramp (n=484)	0 (0)	22 (4.5)	462 (95.5)
Walker or rollator (n=479)	10 (2.1)	44 (9.2)	425 (88.7)
Wheelchair or transport chair (n=530)	0 (0)	57 (10.8)	473 (89.2)

**Table 3:** Mobility-assistive device–specific sample review snippets for each injury type.

Mobility-assistive device	Major injury	Minor injury	Potential future injury
Cane	“[I]t offers no stability. my father’s dr. said stop using immediately. but unfortunately it was after he had a terrible fall, splitting open his head.” “[I] removed the plastic bag and the next thing i knew i was laying on my back hollering, i’ve fallen and i can’t get up! i walked away with a broken rib or two.”	“[W]hen walking out of a restaurant the cane in my left hand snapped in half...i ended up with bruised rib, bruised stomach and head and scratched fingers. i don’t want this to happen to anyone else...very scary for anyone relying on these.” “[W]hen my father sat on it, the plastic clamp like thing that keeps the chair folder [sic] out just gave in!! it’s a miracle that my dad didn’t break something. he landed on his left arm, his fingers were swollen for 4 days.”	“[C]ollapsed due to a broken plastic ring the first time my husband (who weighs less than the advertised maximum for the item) my husband [sic] sat in it and left him sprawled on the floor. fortunately he was not injured.” “[T]he bottom rubber stopper came off while i was walking leaving the metal exposed. i almost fell as it slipped out from under me! do not buy this product!”
Gait or transfer belt	“[T]wice the belts come undone and our family member has been seriously injured!! It’s a horrible feeling when they slip away n fall getting seriously injured w concussion and sprangs [sic] n bruises.”	“When lifting my husband...the transfer gate slide up to his chest. no matter how tight you pull the transfer gate will not stay put. actually hurt my husband. who is a paraplegic.” “[O]nce worn and pulled by the helper it leaves a mark on the waist and the patients complain of pain around the waist.”	“[O]ne of the handles threads snapped within a few minutes of its first use. my father almost fell. this product is dangerously, cheaply made.” “Crappy and unreliable. material is too thin to the point that buckle does not hold adjustment in place. i cannot trust this to secure and hold the elderly person it was bought for.”
Ramp	—^a^	“[T]here are two smooth lines that are very dangerously slippery. knowing that they are there, I’ve slipped many times.” “[T]he ramps are heavy and clumsily to handle. since i am the person who uses the scooter and having to use this has been a big mistake, it has caused my back to hurt.”	“[V]ery little support in the center section so while you are midway with a wheel chair or just walking on it, the ramp bounces and shifts on the step. i don’t feel secure enough to use it with my dad’s wheel chair.” “[I] tried to use this with a lightweight wheelchair and i felt like it was going to buckle/break underneath me...this ramp is suppose [sic] to have a 600lb weight capacity.”
Walker or rollator	“[F]ront right wheel fell off causing my wife to hit her head on a piece of furniture. i had to take her to the emergency room.” “[T]his walker broke apart while my wife was using it. the metal just broke and the walker collapsed. she broke her wrist and hit her head.”	“[T]he legs gave out and my grandma who was sitting on the walker fell back and hit her head on the concrete.” “[T]he back rest connects to the walker via these thick plastic sockets. while my wife was sitting on it both sockets cracked/broke and she fell off the back of the seat hitting her head on a wall. avoid this walker unless you like head injuries.”	“[H]ad a cheap front flat bracket that has a weakness on its bent corner. so if your [sic] sitting on the walker and slide to the right or left there is a chance the bracket crumbling dumping you to the floor...i for one can’t be falling, i am hurt enough and i wouldn’t want my loved ones falling either.” “[Y]esterday my husband was using it and the leg broke in half. thankfully he was not hurt but could have been. it broke completely in two at the weld in the leg. this is a safety issue.”
Wheelchair or transport chair	—	“[B]ack wheels should be farther back. i fell over backwards and was unable to get up by myself. even a little pressure on the handles will cause the chair to tip backward.” “[T]he handles broke off as we were going down a stair step. almost got badly hurt. the pusher fell on top of the patient and the patient’s head hit the last step. very dangerous.”	“[U]nfortunately, when my mom sits down in it, it rocks backward. She’s afraid to use it for fear of flipping over backward. sending it back as it’s unusable if it’s unsteady.” “[T]he brakes shouldn’t be relied on. if you plan to use it in a hilly area, don’t count on them to make much of a difference...they wouldn’t work on a hill or on a long slope.”

^a^Not available.

### Injury Pathway Distributions

Injury pathway by mobility-assistive device–type distributions and totals are displayed in Table 4, and sample cases for each pathway are presented in Textbox 1. Canes had the highest *Critical component breakage or decoupling* rate (240 mentions per 10,000 reviews, 2.4%), closely followed by wheelchairs or transport chairs (222 mentions per 10,000 reviews, 2.22%). Gait or transfer belts had the (substantially) highest *Unintended movement* rate (184 mentions per 10,000 reviews, 1.84%), the next highest being walkers or rollators (101 mentions per 10,000 reviews, 1.01%).

*Instability* rates were highest among canes (180 mentions per 10,000 reviews, 1.8%) and ramps (129 mentions per 10,000 reviews, 1.29%), with users often reporting that they felt unsure about the item’s (ramp’s) ability to securely support them because it felt wobbly or unsteady. *Poor, uneven surface handling* was (mostly) limited to wheeled devices: wheelchairs or transport chairs (46 mentions per 10,000 reviews, 0.46%) and walkers or rollators (26 mentions per 10,000 reviews, 0.26%). Walkers or rollators and canes shared the highest *Trip hazards* rates (15 mentions per 10,000 reviews, 0.15%). As for *Design failure*, canes had the highest review rate (205 mentions per 10,000 reviews, 2.05%), followed by ramps (187 mentions per 10,000 reviews, 1.87%).

From the 6 identified pathways, *Critical component breakage* (855 mentions per 10,000 reviews, 8.55%), *Design failure* (699 mentions per 10,000 reviews, 6.99%), and *Instability* (457 mentions per 10,000 reviews, 4.57%) were found to be the most frequently encountered device failure mechanisms, also accounting for approximately 70% of all major injuries within the data set. Tables S6 and S7 in Multimedia Appendix 1 present key points for each category, rather than review snippets shown in Table 3 and Textbox 1. Moreover, normalized counts regarding device-specific failure mechanisms, discussed in the following section, can be found in Tables S3 and S8 in Multimedia Appendix 1. Table S9 in Multimedia Appendix 1 contains the data set’s nonnormalized (raw) injury-type distributions by mobility-assistive device.

**Table 4:** Injury pathway by mobility-assistive device type per 10,000 reviews.

Injury pathway	Cane, n (%)	Gait or transfer belt, n (%)	Ramp, n (%)	Walker or rollator, n (%)	Wheelchair or transport chair, n (%)
Critical component breakage or decoupling (n=855)	240 (28.1)	123 (14.4)	129 (15.1)	141 (16.5)	222 (26)
Unintended movement (n=434)	30 (6.9)	184 (42.4)	31 (7.1)	101 (23.3)	88 (20.3)
Instability (n=457)	180 (39.4)	0 (0)	129 (28.2)	99 (21.7)	49 (10.7)
Poor, uneven surface handling (n=75)	3 (4)	0 (0)	0 (0)	26 (34.7)	46 (61.3)
Trip hazards (n=41)	15 (36.6)	0 (0)	9 (22)	15 (36.6)	2 (4.9)
Design failure (n=699)	205 (29.3)	86 (12.3)	187 (26.8)	98 (14)	123 (17.6)

**Sample review snippets for each injury pathway.**

**Critical component breakage or decoupling**

- “[T]he handle broke off with the brake assembly causing her to fall. she is now in a recovery/rehabilitation center.” (Product type: walker or rollator; injury type: major injury)
- “[O]ne of the spring loaded buttons that holds the legs in position did not hold and the leg collapsed and i fell breaking a couple of ribs. thank god i didn’t break anything else.” (Product type: walker or rollator; injury type: major injury)
- “[W]e were going down some stairs and when I leaned the chair back the handles snapped dropping both of us down the stairs.” (Product type: wheelchair or transport chair; injury type: minor injury)
- “[S]eat broke attempting to use it the first time the plastic ring supporting the seat broke on the first attempted use and the seat failed. i fell on the floor bruising my tail bone.” (Product type: cane [with seat]; injury type: minor injury)
- “[T]hree months into use, when it broke, nearly sending me to the pavement! one of the metal pegs...sheared off.” (Product type: cane; injury type: potential future injury)

**Unintended movement**

- “[T]he plastic wheels went sliding away from me yet again. just home from the emergency room with a broken left arm above the elbow...two fingers broken badly bruised hips...i would like to go to sleep until i am healed from this horrendous prices [sic] tragedy.” (Product type: walker or rollator; injury type: major injury)
- “[M]y elderly aunt and it is cheap and slipped out from under her. she fell and broke her hip...she was in the hospital for almost a month.” (Product type: walker or rollator; injury type: major injury)
- “[S]uddenly the wheelchair became crazy, drove out by itself and ran into a person before i stopped it...it was scary...think about if i run into kids or right into traffic.” (Product type: wheelchair or transport chair; injury type: minor injury)
- “[I] put it a bit lose [sic] and it rose up to her breast and between me and my sister couldn’t move my mom. after, i made it so tight that she yelped when i buckled it, it still rose up and went under her breast and once again hurt her.” (Product type: gait or transfer belt; injury type: minor injury)
- “[H]owever, i wish it had a rubberized pad to keep from slipping on it. the aluminum tends to get slippery, so watch yourself!” (Product type: ramp; injury type: potential future injury)

**Instability**

- “[I] bought this small walker for my 89 year old 115 pound mother...the walker tipped over and she fell and broke her hip. this product is not strong, stable or well balanced and is very dangerous.” (Product type: walker or rollator; injury type: major injury)
- “not safe...can cause falls-doesn’t feel stable...my dad and a good friend i bought this for stepped on the legs...my dad didn’t fall, but my friend did and cracked her head open on the corner of a wall and has 16 staples.” (Product type: cane; injury type: major injury)
- “[I]t’s a very dangerous equipment...i used it for about two weeks, i tumbled from it twice, because it’s extremely unstable, as you slightly lean forward, it will flips [sic] over.” (Product type: wheelchair or transport chair; injury type: minor injury)
- “[I] walked on a hill and fell 3x. it tips over easily if all 4 legs are not on a flat surface. this cane is a disaster for me.” (Product type: cane; injury type: minor injury)
- “[I]t didn’t lay right as a ramp. when you stood at the top of the ramp the bottom part lifts off the ground.” (Product type: ramp; injury type: potential future injury)

**Poor, uneven surface handling**

- “[W]heel of this walker got caught on a transition from carpet to tile and flipped my grandma over and she hit her head and had to go to the er.” (Product type: walker or rollator; injury type: major injury)
- “[1st] day wheels hung up on threshold of door, mom flew over the top and broke her shoulder! wheels are too small!!” (Product type: walker or rollator; injury type: major injury)
- “[I]f you are planning on using this wheelchair outside or if you are a person with both legs amputated, i would not consider buying this wheelchair, if you come across a crack in the sidewalk this wheelchair will tip on you. i learned the hard way.” (Product type: wheelchair or transport chair; injury type: minor injury)
- “[T]hese tires vibrated the chair significantly over exposed aggregate walkways...this vibration was painful for her. i would not recommend this wheelchair for a recovery where vibration is painful to the surgical site.” (Product type: wheelchair or transport chair; injury type: minor injury)
- “[T]he cane just doesn’t provide the proper support. when on uneven surfaces, the bottom simply doesn’t provide the support of a normal cane. i am severely injured with a spinal cord injury...for me, placing it flat on the ground isn’t as easy as i expected on uneven surfaces.” (Product type: cane; injury type: potential future injury)

**Trip hazards**

- “This walker is dangerous i have tripped over the wheel several times last week i tripped on the wheel and fell i have a bad fracture on my toe and foot.” (Product type: walker or rollator; injury type: major injury)
- “[T]he cane is worthless i tripped on it and broke my right ankle.” (Product type: cane; injury type: major injury)
- “[W]alkers rear wheels are so large they protrude so far back, user can trip very easily. ’ve caught my foot, tripped and fallen at least 5 times already. hard to get used to.” (Product type: walker or rollator; injury type: minor injury)
- “[I] had the hurrycane less then [sic] 10 mins before i tripped over the base causing the cane to collapse on it’s self [sic] and causing me to hit the ground.” (Product type: cane; injury type: minor injury)
- “[T]he hinges on the ramp that allow it to fold up do not recess into the seam once the ramp is opened. the hinges stick up and become trip hazards. i bought the ramp for my elderly father who has trouble navigating steps. he would have tripped on those hinges if he tried to walk up the ramp.” (Product type: ramp; injury type: potential future injury)

**Design failure**

- “[G]randmother…recently fell in the hallway and fractured her pelvis using it. she accidentally pressed one or both of the triggers causing it to fold while using it.” (Product type: walker or rollator; injury type: major injury)
- “[T]he grip has a hard rubber bump that hit right in the center of my palm and transferred the full force of the cane hitting the ground directly through my palm into my wrist. there was none of the shock absorption...it became so painful that i have thrown these away, something i almost never do.” (Product type: cane; injury type: minor injury)
- “[Two] months later it will not stand by itself without my doing a lot of adjusting. it falls over, i have to pick it up and when i bend to pick it up, i frequently fall.” (Product type: cane; injury type: minor injury)
- “[I] assumed the buckle would be located in the middle. it is not. no matter how we tried to adjust it the buckle ends up under my breast making lifting painful.” (Product type: gait or transfer belt; injury type: minor injury)
- “[H]eavy duty, but it’s difficult to undo, would be a big problem in an emergency.” (Product type: gait or transfer belt; injury type: potential future injury)

## Discussion

### Principal Findings

We created a novel data set of safety concerns from reviews of mobility-assistive devices on Amazon’s US website. The data set provided a better understanding of (1) the contexts and conditions leading to user injury and (2) the severity of injuries associated with these mobility-assistive devices. Prior studies of older adult injuries while using mobility-assistive devices reported incidence rates of ED visits and types of injuries associated with mobility-assistive device use [18,19]. By contrast, this study provides novel information about pathways to injuries, including device critical component failures; unintended device movement; poor, uneven surface handling; instability; and trip hazards. Contrary to existing databases such as NEISS and MAUDE, our data set provides a unique consumer-focused perspective on user injury, emphasizing the context and manner in which the devices failed, while also providing basic information regarding injury (none, potential, minor, or major). Demonstrating that timely information can be extracted from online reviews, which are readily available and likely more frequently used than government voluntary reporting systems (owing to ease-of-use reportability) by consumers seeking to report product malfunctions.

Our findings suggest that many mobility-assistive device injuries may be preventable through patient and caregiver education about how to evaluate new and existing equipment for potential future injuries.

We found gait or transfer belts to have an unusually high rate of injuries linked to *Unintended movement*. Reviews generally described the belt slipping through the buckle or sliding up to the user’s chest, resulting in harm to the user. The contents of the reviews provide evidence that gait belt material should be antislip and that buckling systems may need to be redesigned to ensure that belts remain securely fastened. These findings align with those of nursing assistant gait belt reports in Garg et al [20], in which concerns about the belts’ tendency to slip up on the patient were expressed. Walkers or rollators also had relatively high *Unintended movement* rates because users regularly reported that these had faulty brakes, which were incapable of completely stopping device movement, or wheels (especially plastic) with poor traction, placing the older adult at increased fall risk.

Beyond weak braking systems, reviews of walkers or rollators also revealed that some were alarmingly susceptible to *Critical component breakage or decoupling*, having the third highest rate behind that of wheelchairs or transport chairs and canes. Reviews of canes identified bases, handles, and seats (for canes with attached seats) as frequent component breakage locations, with breakage often occurring while the device was being used. Walker and wheelchair user reviews reported similar component breakage issues for both product categories, with mentions of handles or frames snapping and seats failing being fairly common (paralleling canes), in addition to wheels or legs falling off or breaking and screws or nuts coming loose. Given that older adults physically rely upon these devices, repeated mentions of primary user-support–component breakage (handles and seats), as well as critical device-support–location breakage (cane bases, walker legs and wheels, and wheelchair wheels) are extremely worrisome. Furthermore, it should be noted that *Critical component breakage or decoupling* accounted for nearly half of all major injuries within the data set, indicating that re-examination (in light of component breakage) of these mobility-assistive devices may be necessary because serious injuries seem to be occurring because of defective items (ie, sudden breakage), rather than user misuse. Hence, although several studies [21-23] have explored the benefits of adopting such devices during late adulthood, more research is still needed to continue improving the design of these mobility-assistive aids [19].

Wheelchairs or transport chairs and walkers or rollators also lead *Poor, uneven surface handling* counts, with reviews from both device categories describing instances in which these devices were incapable of safely crossing over unlevel surfaces such as seams (as small as tile grout), thresholds, and uneven pavement. These reviews provide further evidence of the need for re-examination of these 2 mobility-assistive device categories, especially walkers or rollators, which also accounted for more than three-quarters of all identified major injuries and had the highest *Trip hazards* rates (alongside canes). Stevens et al [19] also emphasized the need to continue improving the design of mobility-assistive devices, particularly walkers, because they were found to be associated with 7 times as many injuries as canes.

With regard to walker or rollator and cane *Trip hazards*, two-thirds of the data set’s walker or rollator cases were attributed to rear wheel or leg placement being too far back, whereas >80% of the *cane* incidents (within the data set) were associated with quad cane use. Albeit easily addressed with simple design modifications, both *Trip hazards* issues stem from the desire to increase stability, meaning that delicate adjustment is necessary, as evidenced in Mortenson et al [24], to maximize the device’s ease of use while avoiding a permanent decrease to desired stability capabilities.

Regarding this, we found the bases of quad and tripod canes to be highly susceptible to instability, comprising more than two-thirds of identified *Instability* cane instances, implying that, contrary to popular belief, larger cane bases do not to lead to increased stability; rather, they seem to generate more instability than traditional canes. Reviews of ramps also regularly expressed that the device felt unstable, describing it as bending, being depressed, or wobbling in the middle when loaded with moderate weight, generating insecurity and at times forcing the user to implement makeshift (and potentially unsafe) solutions to remediate the issue.

Aside from instability, users frequently reported these 2 product categories (canes and ramps) as having design-related issues (*Design failure)*. With regard to ramps, reviews often described them as having sharp edges, increasing the risk of laceration when handling the device and (potentially) aggravating the consequences of a fall injury, or being too steep, leading to increased fall risk and abnormal gait mechanics to control descent. Cane design issues were commonly associated with poor ergonomic handle design, causing pain while in use, and poor self-standing capabilities (quad or tripod canes), forcing users to repeatedly bend down to pick up the device, potentially leading to, or exacerbating existing, back issues.

In support of injury prevention efforts for this population classified as susceptible, our findings may inform educational interventions to inform older adults and their caregivers about safety issues associated with mobility-assistive equipment. Educational interventions would include information about how to select safe mobility-assistive products, how to fit the mobility-assistive device to the user, how to safely use the newly adopted mobility-assistive devices, and how to assess the safety of the product over time. Moreover, beyond our available data, which provide actionable consumer safety information, future use of the presented injury pathway categories may aid businesses and legislators seeking to monitor device safety because these may allow for more specific search parameters and more effective monitoring of frequent device failure mechanisms.

### Limitations

This study includes several limitations. Although the source data span >600,000 reviews and dozens of categories, we focused only on a subset of 1 category (mobility-assistive devices). We analyzed only injury incidents and concerns actively self-reported by older adults and caregivers within online mobility-assistive device reviews on a single major marketplace (Amazon’s US website); products listed by other retailers may yield different findings. As the sample is subject to self-selection bias, results should be taken as *indicative* of injury occurrence, *not representative* of injury occurrence.

We selected Amazon over other retailers because it is one of the largest and most popular online retailers in the United States, containing dedicated assistive device categories offering hundreds of different brands and products. Selecting a smaller retailer would have likely yielded fewer data, with diminished brand and product diversity. However, because we restricted our sample to reviews on Amazon’s US website, the study does not include experiences concerning devices acquired through medical device stores or specialized pharmacies, which may offer device-fitting services and have trained staff to assist in the selection and purchase of the correct device. As Amazon does not provide any of these services, consumers must conduct their own research at the time of purchase, which may result in certain negative experiences owing to poor equipment selection and fit rather than actual equipment malfunction.

Regardless, there may be skew in who is reporting issues via product reviews and thus some underreporting bias; however, our goal was to surface issues that regulators and manufacturers previously were not aware of to focus remediation efforts, rather than the conventional epidemiological concern of quantifying the incidence and distribution of incidents. The manageable volume of substantial issues surfaced by our method means that regulators and manufacturers can manually investigate the veracity of each report, as well as prioritize and plan remediation efforts.

In addition, within the curated data set, less frequently adopted devices, namely, gait or transfer belts and ramps, have considerably smaller sample sizes than categories such as canes and walkers or rollators. Beyond increased bias, this affects study results in that both normalized (Tables 2 and 4) and raw counts (Tables S4 and S5 in Multimedia Appendix 1) may not be indicative of their injury types and pathways. Most importantly, additional product malfunction mechanisms may have remained hidden because of their low popularity, resulting in the study missing critical information concerning both these devices.

Finally, although the large-scale coding efforts resulted in 100,000 coded reviews, because of limited volunteer labor resources, only instances coded initially as “major injury,” “minor injury,” or “potential future injury” (n=6400, 6.4%) by the volunteers were verified by the authors and doctoral student. Future work could explore the possibility of training machine learning classifiers on the data set to use the models to rapidly annotate hundreds of thousands of reviews, which could then be filtered by desired injury and product type and manually analyzed.

### Conclusions

We introduced a novel online review data set containing safety concerns for mobility-assistive devices typically used by older adults. Our findings highlight device-specific contexts within which these devices are harming older adults and indicate that older adults and their caregivers tend to attribute serious injuries to defective items, rather than misuse.

## Appendix

Multimedia Appendix 1 Supplementary material in the form of 1 figure and 9 tables designed to provide further insight into the coding process and data set.

## Abbreviations

CPSC: Consumer Product Safety Commission
ED: emergency department
MAUDE: Manufacturer and User Facility Device Experience
NEISS: National Electronic Injury Surveillance System

## Data Availability

We have made the data set available at Mendeley [25].
