# Supplementary material for: Safety Concerns in Mobility-Assistive Products for Older Adults: Content Analysis of Online Reviews
Source: J Med Internet Res. 2023 Mar 2;25:e42231. doi: 10.2196/42231 (PMC10020910; doi:10.2196/42231)
Supplement: Multimedia Appendix 1 [file jmir_v25i1e42231_app1.docx]

**Multimedia Appendix 1. Supplement**

**Figure S1. Data coding process visualization.**

**Table S1. Phase I & II Tagger Agreement Statistics by Injury Type Category (Numerator = agreements; Denominator = total cases)**

**Table S2. Retained and Removed Product Categories**

**Table S3. Device-specific failure mechanism counts**

**Table S4. Mobility-assistive device by injury type, raw counts**

**Table S5. Injury pathway by mobility-assistive device type, raw counts**

**Table S6. Key points for each injury type**

**Table S7. Key points for each injury pathway**

**Table S8. Injury pathway by mobility-assistive device, injury type normalized counts**

**Table S9. Injury type by mobility-assistive device distribution (raw data)**


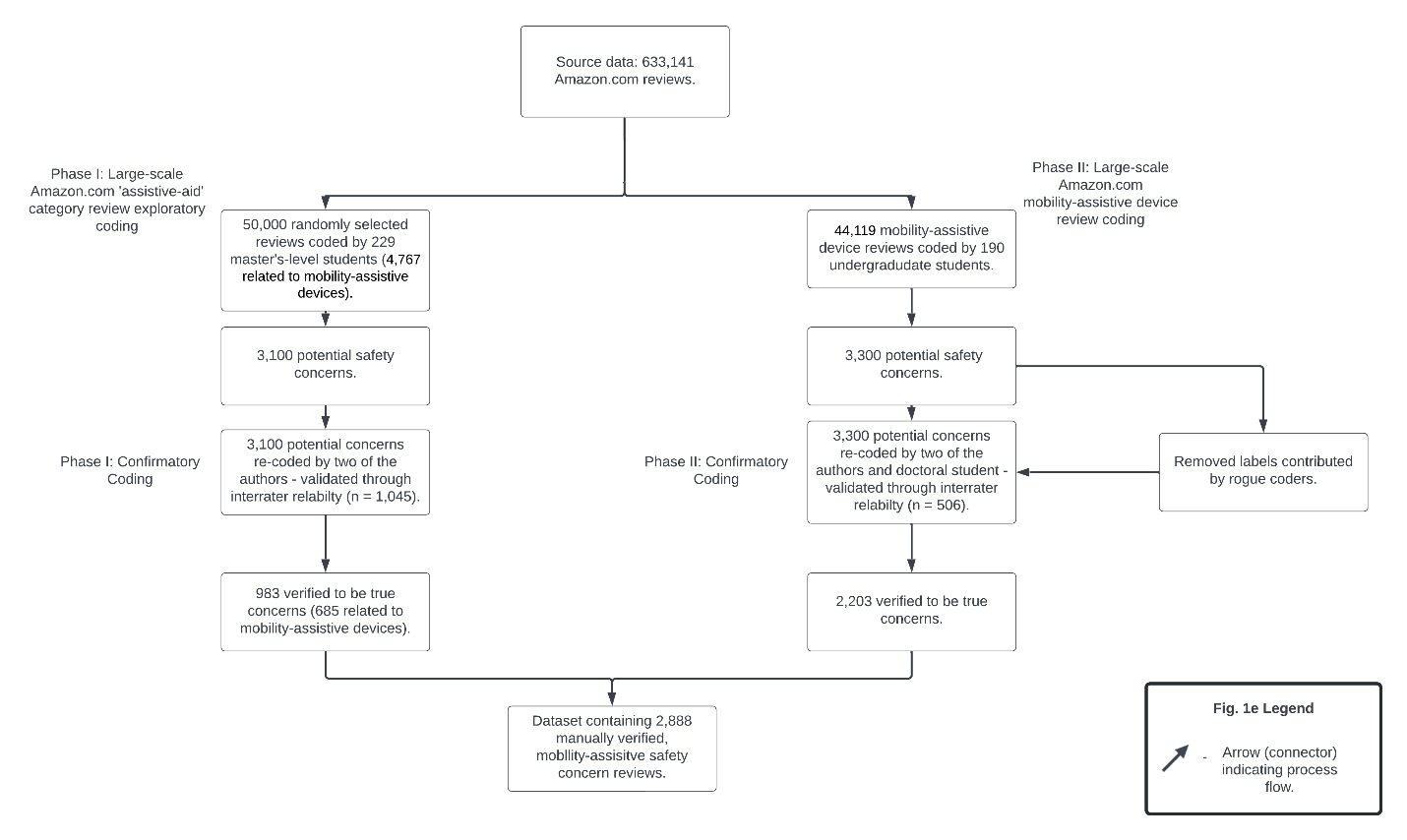


**Figure S1. Data coding process visualization.**

Student-volunteer reviews were randomly selected with replacement and displayed in groups of five. To minimize bias, reviews were blinded so volunteers were not aware of labels assigned by other volunteers. Reviews labelled more than once allowed us to calculate Phase I inter-rater reliability via quadratic weighted Cohen’s Kappa (κ) [15] and validate the large-scale exploratory coding effort. For Phase II we removed unreliable (rogue) coders who marked an unusually high proportion of their reviews as safety concerns, using the following criteria:

- More than 20% of their total labels are safety concerns (potential, minor, or major injury).  In contrast, on average, across all coders, ~12% of reviews (std. dev = 8%) were labelled as safety concerns.
- Labelled more than 2% reviews as ‘Major Injury’. In contrast, on average, across all coders, ~ 0.53% of reviews (std. dev = 1.18%) were labelled as ‘Major Injury’.

For all tagging efforts, in the case of disagreement amongst coders, we allowed the most conservative decision to prevail; that is, an item was regarded as a safety concern if any coder regarded it as a safety concern.

**Table S1. Phase I & II Tagger Agreement Statistics by Injury Type Category (Numerator = agreements; Denominator = total cases)**

|  | **Agreement Counts** | | |
| --- | --- | --- | --- |
| *Phase*  *Injury Type* | **Phase I: Large-scale Exploratory Coding Validation^*^** | **Phase I: Shortlisted Concern Validation Set** | **Phase II: Shortlisted Concern Validation Set** |
|  | *n = 1,258* | *n = 1,045* | *n = 506* |
|  | *Cohen’s κ = 0.59* | *Cohen’s κ = 0.90* | *Cohen’s κ = 0.84* |
| **No Injury** | $\frac{1,068}{1,129}$ | $\frac{51}{62}$ | $\frac{95}{102}$ |
| **Potential Future Injury** | $\frac{35}{84}$ | $\frac{688}{704}$ | $\frac{312}{341}$ |
| **Minor Injury** | $\frac{35}{40}$ | $\frac{234}{255}$ | $\frac{55}{58}$ |
| **Major Injury** | $\frac{2}{5}$ | $\frac{22}{24}$ | $\frac{5}{5}$ |

*We assessed Phase I large-scale efforts inter-rater reliability via quadratic weighted Cohen’s κ [15] because our labelling dimension – injury type – is best characterized as an ordinal variable, where the degree of disagreement between coders fluctuates according to the selected labels. For example, coding a review as “major injury” versus “no injury” would suggest total coder discordance, whereas coding the same review as “major injury” versus “minor injury” would suggest the two coders were close, in that they both agree injury occurred. Hence, since, traditional Cohen’s κ scores do not factor in varying degrees of disagreement between coders, they may not accurately reflect true inter-rater reliability, and weighted κ is a more appropriate measure of agreement. Not seen above is Phase I’s pathway to injury Cohen’s Kappa score, κ = 0.87 [14]. Phase I’s pathway to injury categories were further refined in Phase II by splitting up ‘Design/Material Flaws’ into ‘Critical Component Breakage or Decoupling’ and ‘Design Flaws’.

**Table S2. Retained and Removed Product Categories**

| 1. **Categories Retained** | |  | 1. **Categories Removed** | |
| --- | --- | --- | --- | --- |
| **Mobility related categories** | **Description** |  | **Removed categories** | **Description** |
| Kneewalkers & Scooters | kneewalkers and scooters |  | Toilet Aids & Commodes | raised seats/commodes/toilet stools/urinals/bedpans |
| Rollators & Walkers | rollators/walkers/walker accessories |  | Shower Aids | grab, balance, safety bars/chairs/benches/mats/hair washing basins |
| Canes | canes and hiking sticks |  | Bedroom & Daily Living | overbed tables/rails/ low strength assists/step stools |
| Crutches | crutches/hands free crutches/crutch accessories/ankle braces |  | Reaching Aids | grabbers/reach tools |
| Ramps | ramps |  | Pillows, Cushions, Wedges | cushions, wedges, mattresses, memory foam, pillows |
| Wheelchairs & Transport Chairs | wheelchairs/transport chairs |  | Nail Clippers & Hardeners | nail clippers & nail hardeners |
| Car Accessories | car assist devices |  | Magnifiers & Straws | magnifying devices and straws |
| Gaits & Transfer Belts | gaits and transfer belts |  | Hearing Aids | hearing aids/amplifiers/batteries |
|  | |  | Dressing Aids | shoehorns/stocking donners/dressing aids |
|  |  |  | Misc | other/cast protectors/waterproof covers/writing aids/batteries/flashlights |

**Table S3. Device-specific failure mechanism counts**

| **Mobility Device** | **Specific Failure Mechanism** | **Normalized count per 10,000 reviews** |
| --- | --- | --- |
| Canes | Base/handle/seat breakage | 72 |
|  | Poor ergonomic handle design or self-standing capabilities | 146 |
| Gait or transfer belts | Buckle and belt material slippage | 123 |
| Ramps | Bend, depress, or wobble with moderate weight | 71 |
|  | Too steep or sharp edges | 67 |
| Walkers or rollators & wheelchairs or transport chairs | Faulty brakes and poor tire traction | 62 |
|  | Feet/leg/handle/frame/seat breakage | 87 |
|  | Unable to safely cruise over unlevel surfaces | 19 |

**Table S4. Mobility-assistive device by injury type, raw counts**

| **Mobility Device** | **Major Injury** | **Minor Injury** | **Potential Future Injury** | **TOTAL** |
| --- | --- | --- | --- | --- |
| **Cane** | 4 | 99 | 1087 | 1190 |
| **Gait or Transfer Belt** | 1 | 7 | 56 | 64 |
| **Ramp** | 0 | 5 | 104 | 109 |
| **Walker or Rollator** | 17 | 71 | 693 | 781 |
| **Wheelchair or Transport Chair** | 0 | 63 | 521 | 584 |

**Table S5. Injury pathway by mobility-assistive device type, raw counts**

| **Injury Pathway** | **Cane** | **Gait or Transfer Belt** | **Ramp** | **Walker or Rollator** | **Wheelchair or Transport Chair** | **TOTAL** |
| --- | --- | --- | --- | --- | --- | --- |
| Critical Component Breakage or Decoupling | 426 | 20 | 29 | 234 | 247 | 956 |
| Unintended Movement | 52 | 30 | 7 | 160 | 93 | 776 |
| Unstable | 315 | 0 | 29 | 160 | 54 | 278 |
| Poor Uneven Surface Handling | 6 | 0 | 0 | 44 | 54 | 104 |
| Trip Hazard | 28 | 0 | 2 | 24 | 2 | 56 |
| Design Failure | 363 | 14 | 42 | 159 | 134 | 713 |

**Table S6. Key points for each injury type**

| **Mobility Device** | **Major Injury** | **Minor Injury** | **Potential Future Injury** |
| --- | --- | --- | --- |
| **Cane** | -Caused by cane instability and tripping/stepping on cane legs/base. | -Largely caused by seat malfunctions in canes with seat attachments (plastic ring holding seat together giving away).  -Quad-based cane handle design also frequently caused user hand pain. | -Largely attributed to product malfunctions that did not result in injury.  -Part breakage (handle, cane base).  -Cane instability while using.  -Tri and Quad-based canes failing to stand up on their own. |
| **Gait or Transfer Belt** | — | -Generally attributed to belt material being hurting the patient (cutting them, leaving marks). | - Largely attributed to the belt not staying in place and slipping up towards the patient’s chest while using.  -Also attributed to fraying/unthreading belt material and poor buckle quality (plastic). |
| **Ramp** | — | -Caused by poor ramp handling design (sharp edges), leading to hand injuries when moving. | -Caused by pin and/or velcro strap breakage, compromising ramp integrity.  -Also frequently attributed to ramp instability (wobbly, bendy) while used. |
| **Walker or Rollator** | -Generally caused by part breakage, particularly the wheels, while in use.  -Also caused due to walker wheels getting “stuck” while crossing small thresholds. | -Poor backrest and handle design caused a large percentage of minor injuries.  -Part breakage also accounted for a large number of minor injuries (seat, legs, wheels). | -Largely attributed to part breakage that did not result in injury, walker brake failure resulting in unintended movement, and walker instability. |
| **Wheelchair or Transport Chair** | — | -Largely attributed to poor overall chair design, causing the user pain while sitting in it (especially backrest design). | -Generally attributed to part breakage, specifically wheels and handles.  -Also frequently caused by brake failure, resulting in unintended movement. |

**Table S7. Key points for each injury pathway**

|  | **Key Points** | | | | |
| --- | --- | --- | --- | --- | --- |
| **Injury Pathway** | **Cane** | **Gait or Transfer Belt** | **Ramp** | **Walker or Rollator** | **Wheelchair or Transport Chair** |
| Critical Component Breakage or Decoupling | -Largely caused by handle, base, and seat breakage/detachment (in canes with seats). | -Generally caused by belt material fraying/unthreading and plastic buckle breakage. | -Largely attributed to pin and velcro breakage. | -Generally caused by wheel, leg, handle, and screw/nut breakage/detachment. | -Largely attributed to wheel and handle breakage. |
| Unintended Movement | -Attributed to slippery cane tips and slick handles causing unwanted hand movement. | -Attributed to the belt sliding up towards the patient’s chest while in use. | — | -Largely attributed to brake failure. | -Largely attributed to brake failure. |
| Unstable | -Usually associated with quad-based canes. | — | -Generally attributed to poor construction material, resulting in a ‘bendy’ and ‘wobbly’ ramp | -Often associated with wheels being wobbly, handle placement being too low, and the walker being too light. | -Usually attributed to wheelchair tipping while in use. |
| Poor Uneven Surface Handling | -Attributed to the cane base ‘catching’ onto the ground. | — | — | -Frequently caused by the walker wheels getting ‘caught’ in small cracks or thresholds. | -Frequently caused by the wheelchair wheels getting ‘caught’ in small cracks or thresholds. |
| Trip Hazard | -Largely caused by users tripping or stepping on quad-based cane bases. | — | -Generally attributed to protruding hinges on the ramp’s surface. | -Often attributed to wheel/leg placement, particularly rear wheels/legs being placed too far back. | — |
| Design Failure | -Most frequently attributed to quad/tri-based canes failing to stand up on their own and poor ergonomic handle design. | -Often caused by belt material quality (stretches, harms patient, gets stuck in buckle). | -Largely attributed to sharp edges around the ramp, and (to a lesser extent) being too steep. | -Often attributed to one of the following: absence of brakes, brakes getting stuck, no brake lock, and poor wheel design (too large, too small, plastic). | -Generally caused by handbrake absence and poor arm/foot rest design. |

—: not available.

**Table S8. Injury pathway by mobility-assistive device, injury type normalized counts**

| **Product Type**      **Injury Pathway** | **Cane** | | | **Gait/Transfer Belt** | | | **Ramp** | | | | | **Walker or Rollator** | | | | | **Wheelchair or Transport Chair** | | | | |  |  |
| --- | --- | --- | --- | --- | --- | --- | --- | --- | --- | --- | --- | --- | --- | --- | --- | --- | --- | --- | --- | --- | --- | --- | --- |
|  |  |  |  |  |  |  |  |  |  |  |  |  |  |  |  |  |  |  |  |  |  |  |  |
|  |  |  |  |  |  |  |  |  |  |  |  |  |  |  |  |  |  |  |  |  |  |  |  |
|  | Major Injury | Minor Injury | Potential Future Injury | Major Injury | Minor Injury | Potential Future Injury | Major Injury | | Minor Injury | | Potential Future Injury | Major Injury | | Minor Injury | | Potential Future Injury | Major Injury | | Minor Injury | | Potential Future Injury |  | |
| Critical Component Breakage or Decoupling | 1 | 21 | 219 | 6 | 0 | 117 | 0 | 0 | | 129 | | 4 | 10 | | 129 | | 0 | 16 | | 208 | |  |  |
| Trip Hazard | 1 | 2 | 14 | 0 | 0 | 0 | 0 | 0 | | 9 | | 1 | 2 | | 12 | | 0 | 0 | | 2 | |  |  |
| Poor Uneven Surface Handling | 0 | 0 | 3 | 0 | 0 | 0 | 0 | 0 | | 0 | | 2 | 4 | | 21 | | 0 | 4 | | 45 | |  |  |
| Design Failure | 0 | 24 | 181 | 0 | 25 | 61 | 0 | 18 | | 169 | | 1 | 17 | | 80 | | 0 | 26 | | 95 | |  |  |
| Unintended Movement | 0 | 0 | 29 | 0 | 18 | 166 | 0 | 4 | | 27 | | 1 | 4 | | 93 | | 0 | 5 | | 80 | |  |  |
| Unstable | 1 | 9 | 168 | 0 | 0 | 0 | 0 | 0 | | 129 | | 2 | 6 | | 90 | | 0 | 6 | | 43 | |  |  |

**Table S9. Injury type by mobility-assistive device distribution (raw data)**

| Product Type / Injury Type | Cane | Gait or Transfer Belt | Ramp | Walker or Rollator | Wheelchair or Transport Chair | Total |
| --- | --- | --- | --- | --- | --- | --- |
| Major Injury | 18% | 5% | 0% | 77% | 0% | 100% |
| Minor Injury | 40% | 3% | 2% | 29% | 26% | 100% |
| Potential Future Injury | 44% | 2% | 4% | 28% | 21% | 100% |
